# Pre- and post-diagnosis body weight trajectories in patients with localized renal cell cancer

**DOI:** 10.1007/s10552-024-01957-2

**Published:** 2025-01-06

**Authors:** Alina Vrieling, Linnea T. Olsson, Guyon Kleuters, Jake S. F. Maurits, Katja Aben, J. P. Michiel Sedelaar, Helena Furberg, Lambertus A. L. M. Kiemeney

**Affiliations:** 1 IQ Health Science Department, Radboud University Medical Center, Nijmegen, The Netherlands; 2 Department of Epidemiology, University of North Carolina at Chapel Hill, Chapel Hill, NC USA; 3 Department of Research and Development, Netherlands Comprehensive Cancer Organisation, Utrecht, The Netherlands; 4 Department of Urology, Radboud University Medical Center, Nijmegen, The Netherlands; 5 Department of Epidemiology and Biostatistics, Memorial Sloan Kettering Cancer Center, New York, NY USA

**Keywords:** Carcinoma, Renal cell, Obesity paradox, Body weight changes, Cancer survivors

## Abstract

**Purpose:**

Obesity in mid-life is a well-established risk factor for developing renal cell carcinoma (RCC); however, patients with RCC who are obese at the time of diagnosis have more favorable survival outcomes. To get better insight into the obesity paradox and determine the extent to which weight around diagnosis is stable, we examined pre- and post-diagnosis weight changes in patients with localized RCC.

**Methods:**

We included 334 patients with localized RCC from the prospective cohort ReLife who self-reported body weight at multiple time points ranging from 2 years before to 2 years after diagnosis. Multivariable linear mixed-effects regression models were used to compare weight at each timepoint to weight at diagnosis for the overall study population, as well as stratified by BMI at diagnosis, tumor stage, and tumor grade.

**Results:**

Most patients were classified as overweight (38.3%) or obese (29.6%) at diagnosis. Overall, patients experienced on average 1.45 kg (95% confidence interval (CI) 0.84, 2.06) weight loss in the 2 years before diagnosis. Pre-diagnosis weight loss was higher in patients who were non-obese at diagnosis, and who presented with higher tumor stage and grade. On average, pre-diagnosis weight loss was at least partially regained within two years after diagnosis.

**Conclusion:**

Patients who were non-obese and patients with higher stage and grade tumors had higher pre-diagnosis weight loss, which was at least partially regained after treatment. These patterns suggest there are subgroups of patients with localized RCC who experience disease-related weight loss, which could contribute to the obesity paradox.

**Supplementary Information:**

The online version contains supplementary material available at 10.1007/s10552-024-01957-2.

## Introduction

Renal cell carcinoma (RCC) accounts for approximately 90% of all kidney cancers [1]. Obesity in mid-life is an established risk factor for the development of RCC [2]. Counterintuitively, in clinical studies among patients with RCC, individuals with obesity at the time of diagnosis experience more favorable survival outcomes compared to individuals with normal weight [3, 4]. This phenomenon is known as the obesity paradox [5].

Various hypotheses have been proposed to explain why higher body mass index (BMI) at diagnosis is associated with lower mortality in clinical studies among patients with RCC. Methodological explanations for the obesity paradox include unaddressed confounding, selection bias, and the use of BMI as a measure of obesity [5]. The obesity paradox may also be driven by differences in tumor biology. Prior studies show that patients with RCC who are normal weight at diagnosis present with higher tumor stage and grade than patients who are obese [6, 7]. Molecular studies also suggest that RCC tumors of patients with normal weight at diagnosis are more aggressive than tumors of patients with obesity due to differences in gene expression of metabolic and fatty acid genes [7–11]. Reverse causation could also contribute to the obesity paradox if patients with normal weight have experienced weight loss prior to diagnosis. Thus, weight at time of diagnosis may not accurately reflect a patient’s average, pre-diagnosis adult weight [5].

Only few prior studies have evaluated weight change in relation to clinical outcomes in patients with localized RCC and primarily focused on pre-diagnosis weight loss [12–14]. In this study, we assessed weight at multiple timepoints ranging from two years before to two years after diagnosis and evaluated whether weight changes differed according to BMI at the time of diagnosis, tumor stage, and grade.

## Methods

We used data from the ReLife study, a prospective cohort study of patients with newly diagnosed localized RCC, as described in detail elsewhere [15]. The study was approved by the Committee for Human Research region Arnhem-Nijmegen (CMO 2016–3078). The study involved 18 hospitals in the Netherlands, and eligible patients were identified through the Netherlands Cancer Registry (NCR) from January 2018 to June 2021. Eligible patients were between 18 and 75 years of age, diagnosed with primary stage I–III RCC, and underwent a radical or partial nephrectomy or an ablation. Patients with a prior cancer diagnosis within 5 years before their RCC diagnosis or those with metastatic RCC were not eligible. Patients were invited to participate in the study approximately 10 weeks after treatment; those who agreed provided written informed consent. Of the 837 eligible patients, 368 agreed to participate (response rate of 44%) [15]. Relative to non-responders, participants were more likely to be female, but were similar in terms of age, tumor stage, grade, tumor morphology, and treatment type [15]. Data on weight and height were not available for non-responders. The date of the surgical or ablation treatment is considered the date of diagnosis. For the current study, 334 patients with data from medical records on body weight within 3 months prior to diagnosis were included. The median time from recorded weight to diagnosis was 13 days (interquartile range 1–25 days).

### Weight and height

**Fig. 1:**
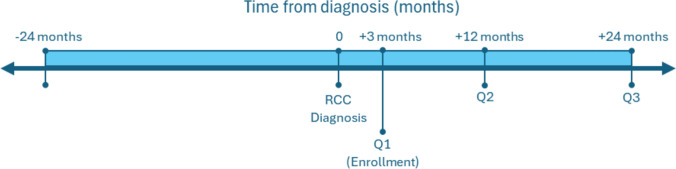
Study timeline Participants were enrolled approximately three months after renal cell cancer (RCC) diagnosis. Data on participants’ weights were collected from questionnaires at three months (Q1), one year (12 months, Q2), and two years (24 months, Q3) after diagnosis. Weight at diagnosis was abstracted from electronic medical records by data managers at the Netherlands Cancer Registry. Weight at two years prior to diagnosis (−24 months) was retrospectively reported by participants in Q1

Participants completed self-administered questionnaires either online or on paper, at 3 months (Questionnaire 1; Q1), 1 year (Questionnaire 2; Q2), and 2 years (Questionnaire 3; Q3) after diagnosis (Fig. 1). Out of the 334 participants, 331 completed Q1, 282 Q2, and 249 Q3. Dropout rate was slightly higher for patients with advanced cancer stage (Stage III), higher tumor grade (Grade 3 and 4), and lower educational level (Suppl. Table 1). Notably, only 11 (3%) patients died during study follow-up.

Q1 collected information on height and on body weight 2 years before diagnosis and 3 months after diagnosis, Q2 on weight 1 year after diagnosis, and Q3 on weight 2 years after diagnosis. Weight at diagnosis was collected from the medical records by data managers of the NCR. BMI at diagnosis was calculated in kg/m^2^ and categorized into four groups: underweight (≤ 18.5), normal weight (18.5–25), overweight (25- ≤ 30), and obese (> 30). As only one patient was underweight, this patient was included in the normal weight category for all analyses.

### Clinical data

Tumor and clinical characteristics were abstracted from medical records by data managers at the NCR and included date of diagnosis, clinical and post-surgical TNM stage, Fuhrman or International Society of Urological Pathologists (ISUP) tumor grade, morphology, and type of treatment.

### Covariates

Information about patient characteristics was collected in Q1. Participants reported their age at diagnosis, biological sex, highest level of education, and cigarette smoking status at diagnosis. Information on 14 comorbidities was collected using an adapted version of the Comorbidity Questionnaire [16].

### Statistical analysis

Distributions of baseline characteristics were calculated for study participants overall and stratified by BMI classification at diagnosis. Mean body weights at each timepoint from 2 years before diagnosis to 2 years after diagnosis were calculated for the total study population and stratified by BMI classification at diagnosis, tumor stage, and tumor grade.

Linear mixed-effects regression models were constructed to compare the weight at each timepoint to the weight at diagnosis. Multivariable models adjusted for age (mean-centered), sex, and cigarette smoking status were used to calculate β coefficients and 95% confidence intervals (CI). Time was included as a categorical fixed variable in all models. First, an overall model was created to investigate changes in weight across the whole study population. Second, models were stratified by categorical variables (BMI at diagnosis, tumor stage, and grade) to investigate whether weight changes differed according to these subgroups. Third, interaction terms between time and BMI, tumor stage, or grade were added to the models to estimate whether the differences in weight between each timepoint and diagnosis varied over levels of these categorical variables. Lastly, models for BMI categories at diagnosis were further stratified by tumor stage (I vs. II-III) and grade (1–2 vs. 3–4) to investigate whether weight change within BMI categories differed according to these subgroups, and interaction terms between time and stage or grade were also included. In a sensitivity analysis, we also investigated whether weight changes differed according to sex but no differences were found (data not shown).

P-values of less than 0.05 were regarded statistically significant. R version 4.1.3 (2022–03-10) was used for statistical analysis (i.e. lme4, lmerTest, emmeans) [17–19].

## Results

Demographic and clinical characteristics for the total study population and stratified by BMI at diagnosis are presented in Table 1. In the overall cohort, the average age at diagnosis was 62.3 (± 9.1) years and 70.1% of patients were male. The average BMI at diagnosis was 27.9 (± 5.0) kg/m^2^, with 38.3% of patients being overweight and 29.6% obese. Most patients were diagnosed with stage I (65.3%) and grade 2 (50.9%) disease. About half of the patients were former smokers.

**Table 1:** Baseline characteristics of patients with localized RCC included in the ReLife study for the overall study population and by BMI category at diagnosis

	All patients	Normal weight	Overweight	Obese
	*N* = *334*	*N* = *107*	*N* = *128*	*N* = *99*
Characteristics	Mean (SD) or N (%)			
Age at diagnosis (years)	62.3 (9.1)	62.9 (10.1)	61.7 (8.8)	62.5 (8.3)
Sex				
Male	234 (70.1)	75 (70.1)	98 (76.6)	61 (61.6)
Female	100 (29.9)	32 (29.9)	30 (23.4)	38 (38.4)
Educational level^a^				
Low	134 (40.1)	34 (31.8)	55 (43.3)	45 (45.5)
Medium	105 (31.4)	30 (28.0)	38 (29.9)	37 (37.4)
High	92 (27.5)	42 (39.3)	34 (26.8)	16 (16.2)
Missing	3 (0.9)	1 (0.9)	1 (0.8)	1 (1.0)
Cigarette smoking status				
Never	126 (37.7)	46 (43.0)	49 (38.3)	31 (31.3)
Former	163 (48.8)	51 (47.7)	60 (46.9)	52 (52.5)
Current	42 (12.6)	9 (8.4)	18 (14.1)	15 (15.2)
Missing	3 (0.9)	1 (0.9)	1 (0.8)	1 (1.0)
Comorbidity				
0	50 (15.0)	22 (20.6)	22 (17.2)	6 (6.1)
1	74 (22.2)	28 (26.2)	28 (21.9)	18 (18.2)
≥ 2	207 (62.0)	56 (52.3)	77 (60.2)	74 (74.7)
Missing	3 (0.9)	1 (0.9)	1 (0.8)	1 (1.0)
Tumor stage				
I	218 (65.3)	74 (69.2)	82 (64.1)	62 (62.6)
II	48 (14.4)	14 (13.1)	19 (14.8)	15 (15.2)
III	68 (20.4)	19 (17.8)	27 (21.1)	22 (22.2)
Tumor grade				
1	46 (13.8)	15 (14.0)	15 (11.7)	16 (16.2)
2	170 (50.9)	50 (46.7)	70 (54.7)	50 (50.5)
3	60 (18.0)	20 (18.7)	25 (19.5)	15 (15.2)
4	20 (6.0)	11 (10.3)	5 (3.9)	4 (4.0)
Missing	38 (11.4)	11 (10.3)	13 (10.2)	14 (14.1)
Morphology tumor^b^				
Clear cell	236 (70.7)	68 (63.6)	97 (75.8)	71 (71.7)
Papillary	44 (13.2)	19 (17.8)	11 (8.6)	14 (14.1)
Chromophobe	22 (6.6)	9 (8.4)	8 (6.3)	5 (5.1)
Other	32 (9.6)	11 (10.3)	12 (9.4)	9 (9.1)
Treatment				
Radical nephrectomy	189 (56.6)	55 (51.4)	76 (59.4)	58 (58.6)
Partial nephrectomy	140 (41.9)	51 (47.7)	51 (39.8)	38 (38.4)
Ablation^c^	5 (1.5)	1 (0.9)	1 (0.8)	3 (3.0)
Weight at diagnosis (kg)	86.9 (16.8)	72.3 (8.3)	85.7 (9.2)	104.3 (15.3)
Height at diagnosis (m)	1.77 (0.09)	1.77 (0.09)	1.79 (0.09)	1.75 (0.09)
BMI at diagnosis (kg/m^2^)	27.9 (5.0)	23.0 (1.5)	27.3 (1.4)	34.0 (3.8)
Weight change from 2 years before diagnosis to diagnosis^d^				
Loss (≥ 5%)	73 (21.9)	29 (27.1)	26 (20.3)	18 (18.2)
Stable (-5 to 5%)	219 (65.6)	72 (67.3)	83 (64.8)	64 (64.6)
Gain (≥ 5%)	35 (10.5)	5 (4.7)	15 (11.7)	15 (15.2)
Missing	7 (2.1)	1 (0.9)	4 (3.1)	2 (2.0)

^a^Low (primary, secondary, and vocational education), medium (intermediate vocational education, higher general secondary education, and pre-university education) and high (university of vocational education and university). ^b^Other morphology consists of renal cell carcinoma not otherwise specified (*n* = 26), adenocarcinoma with mixed subtypes (*n* = 4), and sarcomatoid (*n* = 2) renal cell carcinoma; ^c^Ablation includes radiofrequency ablation (*n* = 3), cryoablation (*n* = 1), and microwave ablation (*n* = 1). ^d^Calculated from weight at 2 years pre-diagnosis and weight at diagnosis

*BMI* Body mass index, *RCC* Renal cell cancer, *SD* Standard deviation

Higher education, no history of smoking, and fewer comorbidities were more common among patients with normal weight at diagnosis. It was also more common for patients with normal weight to have lower stage or higher grade disease and receive partial nephrectomy, but less common to be diagnosed with clear cell RCC.

### Weight changes for the study population overall

**Fig. 2:**
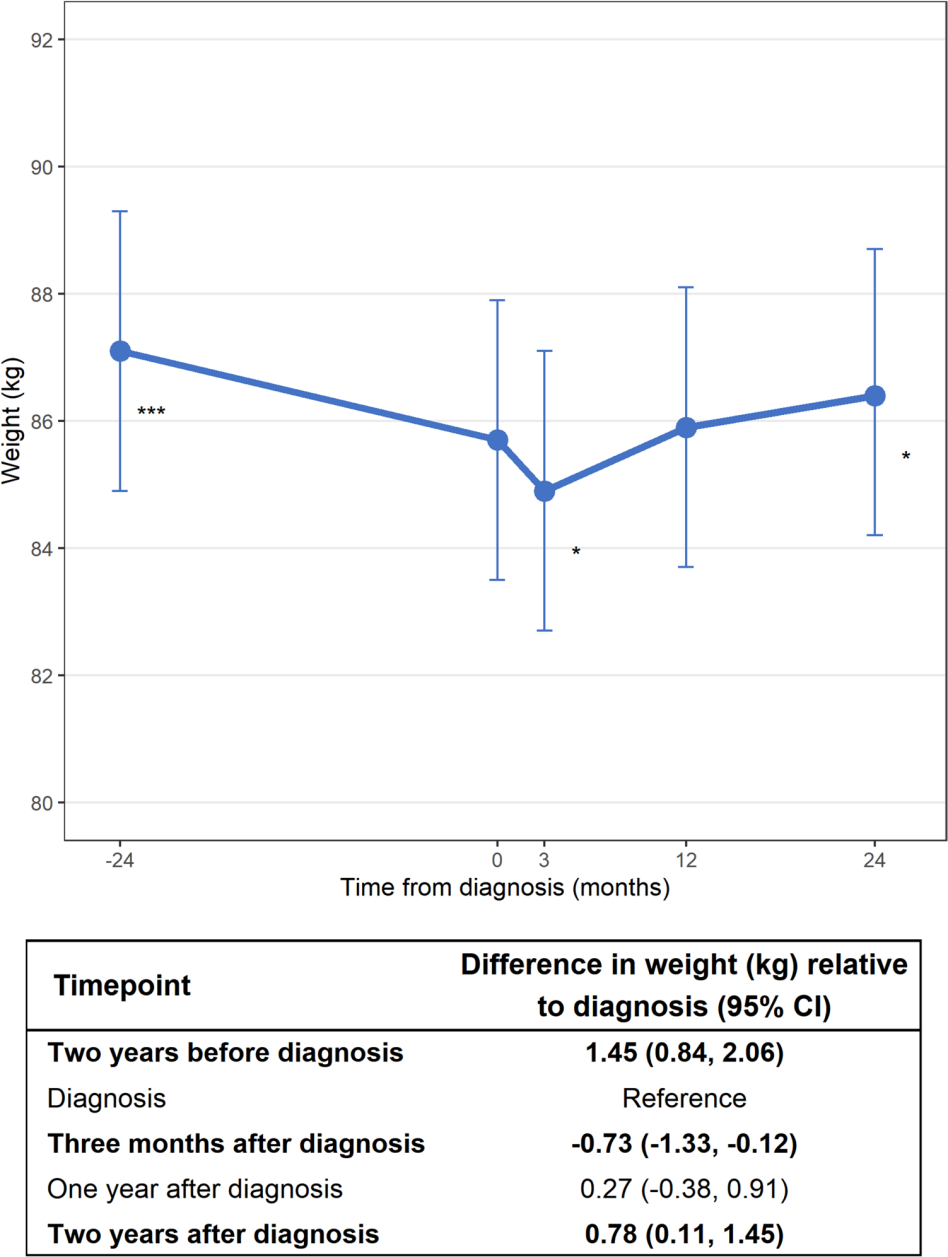
Marginal means and 95% confidence intervals of weight change over time adjusted for age (mean-centered), sex, and smoking status for the total study population of patients with RCC (*N* = 334). Significance levels for the difference between weight at the indicated timepoint compared to diagnosis: ‘*’*p* < 0.05, ‘***’*p* < 0.001

Figure 2 shows adjusted body weights and weight changes from 2 years pre-diagnosis to 2 years post-diagnosis for the total study population. Overall, patients lost a mean of 1.45 kg (95% CI 0.84, 2.06) from 2 years pre-diagnosis to diagnosis. Patients lost a further 0.73 kg (95% CI -1.33, -0.12) from diagnosis to 3 months post-diagnosis, but regained 0.78 kg (95% CI 0.11, 1.45) at 2 years post-diagnosis compared to diagnosis. Mean crude body weights over time are shown in Suppl. Table 2. Some patients shifted across BMI categories in the 2 years prior to diagnosis. Among patients with normal weight at diagnosis, 16.0% were overweight before diagnosis. Among patients with overweight at diagnosis, 9.7% were normal weight and 16.9% were obese before diagnosis. Among patients with obesity at diagnosis, 17.5% were overweight before diagnosis.

### Weight changes for the study population stratified by BMI at diagnosis, tumor stage, and tumor grade

**Fig. 3:**
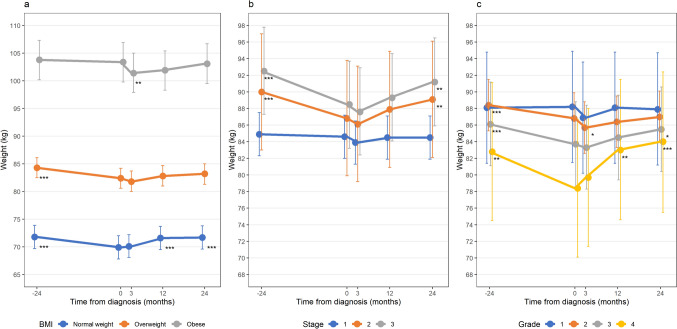
Marginal means and 95% confidence intervals of weight change over time adjusted for age (mean-centered), sex, and smoking status; **a** Stratified by BMI at diagnosis (*N* = 334); Normal weight < 25 kg/m^2^ (*n* = 107), Overweight 25– ≤ 30 kg/m^2^ (*n* = 128), Obese > 30 kg/m.^2^ (*n* = 99); **b** Stratified by tumor stage (*N* = 334; Stage I (*n* = 218), Stage II (*n* = 48), Stage III (*n* = 68). **c** Stratified by tumor grade (*N* = 296); grade 1 (*n* = 46), grade 2 (*n* = 170), grade 3 (*n* = 60), grade 4 (*n* = 20). Significance levels for the difference between weight at the indicated timepoint compared to diagnosis within the same subgroup: ‘*’*p* < 0.05, ‘**’*p* < 0.01, ‘***’*p* < 0.001

Figure 3 shows adjusted body weights, and Table 2 adjusted weight changes, from 2 years pre-diagnosis to 2 years post-diagnosis, stratified by BMI at diagnosis (Fig. 3A), tumor stage (Fig. 3B), and grade (Fig. 3C).

**Table 2:** Difference in weight (kg) for each time point compared to diagnosis with 95% confidence intervals (CIs) among patients with RCC from linear mixed-effect regression models adjusted for age, sex, and smoking status for the total population and stratified by BMI at diagnosis, tumor stage, and tumor grade (marginal means and 95% CIs belonging to this model are shown in Fig. 3)

	Difference in weight (kg)	95% CI	
Normal weight			
2 years before diagnosis	**1.86**	**1.11**	**2.61**
Diagnosis	Reference		
3 months after diagnosis	0.21	–0.54	0.96^a^
1 year after diagnosis	**1.66**	**0.88**	**2.45**^**a**^
2 years after diagnosis	**1.79**	**0.96**	**2.63**^**a**^
Overweight			
2 years before diagnosis	**1.90**	**0.96**	**2.85**^**a**^
Diagnosis	Reference		
3 months after diagnosis	–0.57	–1.50	0.37
1 year after diagnosis	0.45	–0.55	1.46^a^
2 years after diagnosis	0.76	–0.27	1.78
Obese			
2 years before diagnosis	0.38	–1.06	1.83
Diagnosis	Reference		
3 months after diagnosis	**–1.94**	–**3.38**	–**0.50**
1 year after diagnosis	**–**1.50	–3.02	0.02
2 years after diagnosis	**–**0.29	–1.88	1.30
Stage I			
2 years before diagnosis	0.29	–0.43	1.01
Diagnosis	Reference		
3 months after diagnosis	**–**0.70	–1.42	0.03
1 year after diagnosis	**–**0.10	–0.86	0.66
2 years after diagnosis	**–**0.11	–0.90	0.68
Stage II			
2 years before diagnosis	**3.16**	**1.62**	**4.70**^**a**^
Diagnosis	Reference		
3 months after diagnosis	**–**0.71	–2.24	0.82
1 year after diagnosis	1.06	–0.54	2.66
2 years after diagnosis	**2.25**	**0.61**	**3.89**^**a**^
Stage III			
2 years before diagnosis	**4.06**	**2.58**	**5.55**^**a**^
Diagnosis	Reference		
3 months after diagnosis	**–**0.83	–2.29	0.64
1 year after diagnosis	0.86	–0.74	2.46
2 years after diagnosis	**2.74**	**1.03**	**4.44**^**a**^
Grade 1			
2 years before diagnosis	**–**0.14	–1.52	1.24
Diagnosis	Reference		
3 months after diagnosis	**–**1.32	–2.70	0.06
1 year after diagnosis	**–**0.13	–1.57	1.31
2 years after diagnosis	**–**0.29	–1.78	1.20
Grade 2			
2 years before diagnosis	**1.56**	**0.66**	**2.45**
Diagnosis	Reference		
3 months after diagnosis	**–1.14**	–**2.03**	**-0.25**
1 year after diagnosis	**–**0.41	–1.34	0.52
2 years after diagnosis	0.17	–0.80	1.15
Grade 3			
2 years before diagnosis	**2.44**	**1.18**	**3.70**^**a**^
Diagnosis	Reference		
3 months after diagnosis	**–**0.35	–1.60	0.90
1 year after diagnosis	0.81	–0.56	2.17
2 years after diagnosis	**1.83**	**0.42**	**3.25**
Grade 4			
2 years before diagnosis	**4.45**	**1.73**	**7.17**^**a**^
Diagnosis	Reference		
3 months after diagnosis	1.30	–1.42	4.02
1 year after diagnosis	**4.62**	**1.54**	**7.70**^**a**^
2 years after diagnosis	**5.58**	**2.42**	**8.75**^**a**^

^a^Statistically significant difference (*p* < 0.05) in weight change between each timepoint and diagnosis relative to the weight change in reference category (i.e., obese, stage 1, grade 1)

Bold: Statistically significant difference (p<0.05) in weight between each timepoint relative to the weight at diagnosis

*BMI* Body mass index, *CI* Confidence interval, *RCC* Renal cell cancer

Patients with normal weight at diagnosis had a significant pre-diagnosis weight loss (1.86 kg; 95% CI 1.11, 2.61), but showed a significant weight gain at 1 year (1.66 kg; 0.88, 2.45) and 2 years (1.79 kg; 95% CI 0.96, 2.63) post-diagnosis. Patients with overweight had a significant weight loss pre-diagnosis (1.90 kg; 95% CI 0.96, 2.85), but no significant post-diagnosis weight change. In contrast, among patients with obesity no pre-diagnosis weight loss was observed; however, they did exhibit significant weight loss at 3 months post-diagnosis which was partially regained at 2 years post-diagnosis.

Among patients with stage I disease or grade 1 disease, no significant weight changes were observed from 2 years pre-diagnosis to 2-years post-diagnosis. Patients presenting with stage II and III disease and those with grade 2–4 disease had a significant pre-diagnosis weight loss, ranging from 1.56 kg to 4.45 kg, with higher weight loss for more advanced stages and higher grades. At 2 years after diagnosis, there was a significant weight gain for stage II and III and grade 3 and 4 disease.

Weight changes observed were generally higher for patients with normal weight and overweight versus obesity, patients with stage II and III versus stage I disease, and patients with grade 4 versus grade 1 disease (Table 2, Suppl. Table 3).

### Weight changes for patients with normal weight, overweight, and obesity, stratified by tumor stage and grade

Table 3 shows adjusted weight changes in patients with normal weight, overweight, and obesity at diagnosis, stratified by tumor stage and grade. Among patients with stage II or III disease, pre-diagnosis weight loss was observed within all BMI categories. Among patients with stage I disease, only patients with normal weight experienced significant pre-diagnosis weight loss. Both patients with grade 1–2 and grade 3–4 disease who were normal weight and overweight experienced pre-diagnosis weight loss, which was higher for those with grade 3–4 versus grade 1–2 disease. At 2 years post-diagnosis, weight in all groups was at least partially regained.

**Table 3:** Difference in weight (kg) for each time point compared to diagnosis with 95% confidence intervals (CIs) among patients with RCC from linear mixed-effect regression models adjusted for age, sex, and smoking status stratified by BMI at diagnosis, and subsequently stratified by stage and grade

	Total			Stage I			Stage II–III			Grade 1–2			Grade 3–4		
	Difference	95% CI		Difference	95% CI		Difference	95% CI		Difference	95% CI		Difference	95% CI	
BMI category															
*Normal weight*	*N* = *107*			*N* = *74*			*N* = *33*			*N* = *65*			*N* = *31*		
2 years before diagnosis	**1.86**	**1.11**	**2.61**	**1.19**	**0.35**	**2.04**	**3.38**	**1.86**	**4.89**^**a**^	**1.22**	**0.29**	**2.16**	**2.94**	**1.44**	**4.43**^**b**^
Diagnosis	Reference			Reference			Reference			Reference			Reference		
3 months after diagnosis	0.21	–0.54	0.96	–0.05	–0.90	0.80	0.80	–0.71	2.32	-0.50	–1.44	0.44	0.94	–0.56	2.43
1 year after diagnosis	**1.66**	**0.88**	**2.45**	**1.07**	**0.20**	**1.94**	**3.12**	**1.47**	**4.77**^**a**^	0.85	-0.10	1.80	**3.06**	**1.45**	**4.67**^**b**^
2 years after diagnosis	**1.79**	**0.96**	**2.63**	**1.10**	**0.18**	**2.02**	**3.57**	**1.79**	**5.36**^**a**^	0.69	-0.33	1.71	**3.73**	**2.03**	**5.44**^**b**^
*Overweight*	*N* = *128*			*N* = *82*			*N* = *46*			*N* = *85*			*N* = *30*		
2 years before diagnosis	**1.90**	**0.96**	**2.85**	0.38	–0.65	1.40	**4.82**	**2.96**	**6.70**^**a**^	**1.67**	**0.51**	**2.83**	**4.13**	**2.01**	**6.26**^**b**^
Diagnosis	Reference			Reference			Reference			Reference			Reference		
3 months after diagnosis	–0.57	–1.50	0.37	–0.83	–1.85	0.19	–0.09	–1.8492	1.74	–0.81	–1.96	0.34	–0.13	–2.23	1.97
1 year after diagnosis	0.45	–0.55	1.46	–0.27	–1.35	0.82	**1.78**	–**0.23**	**3.80**	0.00	–1.20	1.21	1.46	–1.00	3.92
2 years after diagnosis	0.76	–0.27	1.78	–0.30	–1.39	0.78	**2.96**	**0.83**	**5.08**^**a**^	0.53	–0.70	1.77	1.67	–0.75	4.09
*Obese*	*N* = *99*			*N* = *62*			*N* = *37*			*N* = *66*			*N* = *19*		
2 years before diagnosis	0.38	–1.06	1.83	–0.90	–2.85	1.04	**2.56**	**0.56**	**4.56**^**a**^	0.53	–1.28	2.34	1.16	–1.53	3.84
Diagnosis	Reference			Reference			Reference			Reference			Reference		
3 months after diagnosis	–**1.94**	–**3.38**	–**0.50**	–1.30	–3.24	0.65	–**3.00**	–**4.98**	–**1.02**	–**2.31**	–**4.11**	–**0.50**	–1.05	–3.74	1.63
1 year after diagnosis	–1.50	–3.02	0.02	–1.37	–3.46	0.73	–1.65	–3.69	0.39	–**2.05**	–**3.97**	–**0.14**	–0.23	–3.02	2.55
2 years after diagnosis	–0.29	–1.88	1.30	–1.33	–3.56	0.90	1.11	–0.97	3.19	–1.12	–3.10	0.86	2.56	–0.42	5.53

^a^Statistically significant difference (*p* < 0.05) in weight change between each timepoint and diagnosis relative to the weight change in the stage I patients

^b^Statistically significant difference (*p* < 0.05) in weight change between each timepoint and diagnosis relative to the weight change in the grade 1–2 patients

Bold: Statistically significant difference (p<0.05) in weight between each timepoint relative to the weight at diagnosis

*BMI* Body mass index, *CI* Confidence interval, *RCC* Renal cell cancer

## Discussion

We described changes in body weight from two years pre-diagnosis through two years post-diagnosis in patients with localized RCC, and explored whether weight changes differ according to BMI at diagnosis, tumor stage, and tumor grade. In the overall population, there was an average pre-diagnosis to diagnosis weight loss of 1.45 kg. Pre-diagnosis weight loss was higher for patients with normal weight or overweight at the time of diagnosis compared to patients with obesity, who maintained a stable weight between pre-diagnosis and diagnosis. Patients with higher tumor stage and grade also experienced higher pre-diagnosis weight loss. Within two years post-diagnosis, most patients either partially or fully regained the weight lost pre-diagnosis regardless of their BMI or pathological characteristics at the time of diagnosis. These findings suggest that in certain subgroups of patients with localized RCC, weight is not stable around the time of diagnosis.

Our finding that weight loss prior to diagnosis was higher in patients classified as non-obese than obese is in line with findings from prior retrospective cohort studies in patients with RCC [3, 12]. One of these studies, defining weight loss as a decrease of ≥ 10% body weight in the 6 months before diagnosis, found that pre-diagnosis weight loss was a stronger prognostic factor for RCC-specific mortality (HR 1.75, 95% CI, 1.13–2.73) than BMI at diagnosis [12]. Another study also showed that pre-diagnosis weight loss, defined as an unintended decrease of ≥ 5 pounds/2.3 kg body weight in the 3 months before diagnosis, was associated with increased RCC-specific mortality (HR 7.9, *p* = 0.002) [13]. A recent study suggested that weight loss around diagnosis, and not low BMI at diagnosis itself, was associated with an increased risk of RCC-specific mortality [14]. These findings are largely consistent with the hypothesis that reverse causation may contribute to the obesity paradox.

We found that patients with higher stage and grade tumors experienced more pre-diagnosis weight loss compared to those with the lowest stage and grade. To our knowledge, no prior study has investigated associations between pre-diagnosis weight loss and RCC stage or grade. However, several studies showed that patients who are normal weight at diagnosis are more likely to present with more aggressive RCC tumors based on tumor stage and grade [6, 7] as well as gene expression of metabolic and fatty acid genes [7–11]. Our findings support the hypothesis that weight loss before diagnosis may be related to more advanced tumor characteristics, which appears to be restored once the tumor has been removed.

Our study has several strengths. To our knowledge, this is the first study to describe body weight trajectories from two years pre-diagnosis through two years post-diagnosis in patients with localized RCC. Body weight data collected at multiple time points were analyzed using linear mixed-effects models. This method relies on the assumption that the outcome data is missing at random. Although participants who dropped out were slightly more likely to have an advanced tumor stage (stage III) and higher tumor grade (grade 3 and 4), they did not differ in pre-diagnosis weight loss. This suggests that selective drop-out is unlikely to have influenced our results. Moreover, very few patients died during follow-up thereby minimizing the likelihood for survivor bias.

We acknowledge study limitations. First, the sample size of this study was relatively small for subgroup analyses, which resulted in estimates with wide confidence intervals, particularly for patients with grade 4 disease (*n* = 20). We therefore regard our findings as hypothesis generating. Second, it is not clear whether tumor grade referred to Fuhrman and ISUP grade. Both grading systems were still in use during our recruitment period, and the specific grading system used was not registered by the NCR, which may have affected our categorization of tumor grade [20]. Third, body weight and height were self-reported, introducing the possibility of measurement error. Although cross-sectional data showed that self-reported weight is generally slightly underreported [21], studies with similar demographic characteristics to our study showed good-to-excellent agreement for self-reported and directly measured body weights [22, 23]. Also, the degree of underreporting is likely to be similar at each time point since survey participants were found to have internal consistency in their reporting over time [24]. Thus, changes in weight may be less prone to measurement error than individual weight measurements. Fourth, weight at 2 years before diagnosis was recalled while post-diagnosis weights were collected prospectively. However, agreement for pre-diagnosis weight recalled shortly after diagnosis and directly measured pre-diagnosis weight was also shown to be good-to-excellent [25]. Lastly, we cannot distinguish between intentional and unintentional weight loss.

Nevertheless, our findings highlight that weight at the time of diagnosis is not stable for all patients and should not be regarded as representative of pre-diagnosis weight, particularly for patients classified as non-obese at diagnosis and with advanced pathological tumor characteristics. Future studies on the clinical impact of body size in patients with RCC should move away from analyses relying solely on weight at the time of diagnosis and instead evaluate how body weight trajectories spanning the pre-diagnosis through post-diagnosis interval are associated with survival outcomes. Refining BMI into body composition features would also provide additional insights, as BMI cannot distinguish between the quantity and quality of skeletal muscle and adipose tissues, which exert distinct biologic effects. Furthermore, examining differences in body weight trajectories according to RCC gene expression profiles would provide further insight into whether tumor aggressiveness influences weight change.

In conclusion, our study suggests that weight at the time of RCC diagnosis is not necessarily stable and for some patients is affected by pre-diagnosis weight loss. Pre-diagnosis weight loss was higher in non-obese individuals and in individuals with higher stage and grade tumors but was largely regained after treatment. This raises the possibility that the obesity paradox may be influenced by disease-related weight loss (i.e., reverse causation).

## Supplementary Information

Below is the link to the electronic supplementary material.

Supplementary file1 (DOCX 123 KB)

## Data Availability

The data sets generated during and/or analyzed during the current study are available from the corresponding author on reasonable request.
