# Supplementary material for: Pre- and post-diagnosis body weight trajectories in patients with localized renal cell cancer
Source: Cancer Causes Control. 2025 Jan 6;36(5):497–507. doi: 10.1007/s10552-024-01957-2 (PMC11982074; doi:10.1007/s10552-024-01957-2)
Supplement: Supplementary file 1 — Supplementary file1 (DOCX 123 KB) [file 10552_2024_1957_MOESM1_ESM.docx]

**Supplementary Table 1.** Baseline characteristics of patients with localized RCC included in the ReLife study for the total study population and by availability of body weight data at 1y and 2y after diagnosis.

|  | **All patients** | **Data on weight 1 y after diagnosis** | | **Data on weight 2 y after diagnosis** | |
| --- | --- | --- | --- | --- | --- |
|  | *N = 334* | **Yes**  *N = 282* | **No**  *N = 52* | **Yes**  *N = 249* | **No**  *N = 85* |
| Characteristics | Mean (SD) or N (%) | |  |  |  |
| **Age at diagnosis (years)** | 62.3 (9.1) | 62.5 (8.9) | 61.5 (10.1) | 62.1 (8.9) | 62.9 (9.7) |
| **Sex** |  |  |  |  |  |
| Male | 234 (70.1) | 195 (69.1) | 39 (75.0) | 178 (71.5) | 56 (65.9) |
| Female | 100 (29.9) | 87 (30.9) | 13 (25.0) | 71 (28.5) | 29 (34.1) |
| **Educational level^a^** |  |  |  |  |  |
| Low | 134 (40.1) | 113 (40.1) | 21 (40.4) | 92 (36.9) | 42 (49.4) |
| Medium | 105 (31.4) | 83 (29.4) | 22 (42.3) | 81 (32.5) | 24 (28.2) |
| High | 92 (27.5) | 83 (29.4) | 9 (17.3) | 74 (29.7) | 18 (21.2) |
| Missing | 3 (0.9) | 3 (1.1) |  | 2 (0.8) | 1 (1.2) |
| **Cigarette smoking status** |  |  |  |  |  |
| Never | 126 (37.7) | 107 (37.9) | 19 (36.5) | 96 (38.6) | 30 (35.3) |
| Former | 163 (48.8) | 136 (48.2) | 27 (51.9) | 122 (49.0) | 41 (48.2) |
| Current | 42 (12.6) | 36 (12.8) | 6 (11.5) | 29 (11.6) | 13 (15.3) |
| Missing | 3 (0.9) | 3 (1.1) |  | 2 (1.2) | 1 (1.2) |
| **Comorbidity** |  |  |  |  |  |
| 0 | 50 (15.0) | 43 (15.2) | 7 (13.5) | 32 (12.9) | 18 (21.2) |
| 1 | 74 (22.2) | 64 (22.7) | 10 (19.2) | 59 (23.7) | 15 (17.6) |
| ≥2 | 207 (62.0) | 172 (61.0) | 35 (67.3) | 156 (62.7) | 51 (60.0) |
| Missing | 3 (0.9) | 3 (1.1) |  | 2 (0.8) | 1 (1.2) |
| **Tumor stage** |  |  |  |  |  |
| I | 218 (65.3) | 187 (66.3) | 31 (59.6) | 167 (67.1) | 51 (60.0) |
| II | 48 (14.4) | 42 (14.9) | 6 (11.5) | 39 (15.7) | 9 (10.6) |
| III | 68 (20.4) | 53 (18.8) | 15 (28.8) | 43 (17.3) | 25 (29.4) |
| **Tumor grade** |  |  |  |  |  |
| 1 | 46 (13.8) | 41 (14.5) | 5 (9.6) | 36 (14.5) | 10 (11.8) |
| 2 | 170 (50.9) | 150 (53.2) | 20 (38.5) | 132 (53.0) | 38 (44.7) |
| 3 | 60 (18.0) | 47 (16.7) | 13 (25.0) | 42 (16.9) | 18 (21.2) |
| 4 | 20 (6.0) | 14 (5.0) | 6 (11.5) | 13 (5.2) | 7 (8.2) |
| Missing | 38 (11.4) | 30 (10.6) | 8 (15.4) | 26 (10.4) | 12 (14.1) |
| **Morphology tumor^b^** |  |  |  |  |  |
| Clear cell | 236 (70.7) | 198 (70.2) | 38 (73.1) | 176 (70.7) | 60 (70.6) |
| Papillary | 44 (13.2) | 38 (13.5) | 6 (11.5) | 34 (13.7) | 10 (11.8) |
| Chromophobe | 22 (6.6) | 16 (5.7) | 6 (11.5) | 15 (6.0) | 7 (8.2) |
| Other | 32 (9.6) | 30 (10.6) | 2 (3.8) | 24 (9.6) | 8 (9.4) |
| **Treatment** |  |  |  |  |  |
| Radical nephrectomy | 189 (56.6) | 158 (56.0) | 31 (59.6) | 138 (55.4) | 51 (60.0) |
| Partial nephrectomy | 140 (41.9) | 121 (42.9) | 19 (36.5) | 108 (43.4) | 32 (37.6) |
| Ablation^c^ | 5 (1.5) | 3 (1.1) | 2 (3.8) | 3 (1.2) | 2 (2.4) |
| **Weight at diagnosis (kg)** | 86.9 (16.8) | 86.3 (16.8) | 90.4 (16.5) | 87.1 (17.1) | 86.4 (16.0) |
| **Height at diagnosis (m)** | 1.77 (0.09) | 1.76 (0.09) | 1.78 (0.09) | 1.77 (0.09) | 1.75 (0.09) |
| **BMI at diagnosis (kg/m^2^)** | 27.9 (5.0) | 27.7 (5.0) | 28.6 (4.7) | 27.8 (5.0) | 28.1 (4.8) |
| **BMI category at diagnosis (kg/m^2^)** |  |  |  |  |  |
| Normal weight (≤25) | 107 (32.0) | 93 (33.0) | 13 (25.0) | 77 (30.9) | 29 (34.1) |
| Overweight (25-≤30) | 128 (38.3) | 104 (36.9) | 24 (46.2) | 97 (39.0) | 31 (36.5) |
| Obese (>30) | 99 (29.6) | 84 (29.8) | 15 (28.8) | 74 (29.7) | 25 (29.4) |
| **Weight change from 2 years before diagnosis to diagnosis^d^** |  |  |  |  |  |
| Loss (≥5%) | 73 (21.9) | 63 (22.3) | 10 (19.2) | 56 (22.5) | 17 (20.0) |
| Stable (-5 to 5%) | 219 (65.6) | 184 (65.2) | 35 (67.3) | 160 (64.3) | 59 (69.4) |
| Gain (≥5%) | 35 (10.5) | 28 (9.9) | 7 (13.5) | 28 (11.2) | 7 (8.2) |
| Missing | 7 (2.1) | 7 (2.5) | 0 (0) | 5 (2.0) | 2 (2.4) |

^a^ Low (primary, secondary, and vocational education), medium (intermediate vocational education, higher general secondary education, and pre-university education) and high (university of vocational education and university). ^b^ Other morphology consists of renal cell carcinoma not otherwise specified (n=28), adenocarcinoma with mixed subtypes (n=4), sarcomatoid renal cell carcinoma (n=2), and eosinophilic solid and cystic renal cell carcinoma (n=1); ^c^ Ablation includes of radiofrequency ablation (n=3), cryoablation (n=2), and microwave ablation (n=1). ^d^ Calculated from weight at 2 years pre-diagnosis and weight at diagnosis.

**Supplementary Table 2.** Crude means (± SD) for body weight (kg) over time, stratified by BMI at diagnosis, tumor stage, and tumor grade, among patients with localized RCC.

|  | n | **2 years before diagnosis** | | | **Diagnosis** | | | **3 months after**  **diagnosis** | | | **1 year after**  **diagnosis** | | | **2 years after  diagnosis** | | |
| --- | --- | --- | --- | --- | --- | --- | --- | --- | --- | --- | --- | --- | --- | --- | --- | --- |
|  |  | 327 | | | 334 | | | 331 | | | 282 | | | 249 | | |
| **BMI at diagnosis** |  |  |  |  |  |  |  |  |  |  |  |  |  |  |  |  |
| normal weight (≤25 kg/m^2^) | 107 | 74.2 | ± | 9.1 | 72.3 | ± | 8.3 | 72.5 | ± | 8.6 | 73.7 | ± | 9.2 | 74.1 | ± | 9.3 |
| overweight (25-≤30 kg/m^2^) | 128 | 87.8 | ± | 11.8 | 85.57 | ± | 9.12 | 85.2 | ± | 9.4 | 85.4 | ± | 9.2 | 86.3 | ± | 10.1 |
| obese (>30 kg/m^2^) | 99 | 104.9 | ± | 17.9 | 104.3 | ± | 15.3 | 102.4 | ± | 15.7 | 102.5 | ± | 16.7 | 104.6 | ± | 17.4 |
|  |  |  |  |  |  |  |  | . |  |  |  |  |  |  |  |  |
| **Tumor stage** |  |  | | |  | | |  | | |  | | |  | | |
| I | 218 | 87.3 | ± | 17.1 | 87.0 | ± | 16.7 | 86.3 | ± | 16.5 | 86.0 | ± | 16.4 | 86.4 | ± | 16.4 |
| II | 48 | 89.4 | ± | 19.3 | 86.3 | ± | 17.4 | 85.6 | ± | 16.5 | 88.2 | ± | 16.5 | 90.7 | ± | 18.5 |
| III | 68 | 91.7 | ± | 19.2 | 87.1 | ± | 16.8 | 86.5 | ± | 16.3 | 87.5 | ± | 17.2 | 91.2 | ± | 19.2 |
|  |  |  |  |  |  |  |  |  |  |  |  |  |  |  |  |  |
| **Tumor grade** |  |  |  |  |  |  |  |  |  |  |  |  |  |  |  |  |
| 1 | 46 | 88.0 | ± | 19.4 | 87.5 | ± | 17.9 | 86.8 | ± | 17.3 | 87.1 | ± | 18.0 | 89.0 | ± | 17.3 |
| 2 | 170 | 90.1 | ± | 18.7 | 88.6 | ± | 17.4 | 87.4 | ± | 17.0 | 86.9 | ± | 16.5 | 88.3 | ± | 17.7 |
| 3 | 60 | 86.9 | ± | 14.5 | 84.2 | ± | 13.6 | 83.9 | ± | 14.0 | 84.8 | ± | 15.3 | 85.4 | ± | 16.7 |
| 4 | 20 | 83.3 | ± | 13.4 | 78.8 | ± | 13.3 | 80.1 | ± | 13.0 | 83.9 | ± | 13.8 | 87.2 | ± | 16.1 |
| Missing | 38 | 86.6 | ± | 19.2 | 87.3 | ± | 17.6 | 87.2 | ± | 17.1 | 88.4 | ± | 18.1 | 88.8 | ± | 17.6 |

**Supplementary Table 3.** Differences in weight change (kg) between each timepoint and diagnosis relative to the weight change in the reference group (obesity, tumor stage I, tumor grade 1) among patients with RCC from linear mixed-effect regression models adjusted for age, sex, and smoking status.

|  | Difference in weight change (kg) | 95% CI |
| --- | --- | --- |
| **BMI category** |  |  |
| Obese | Reference | |
| Normal weight |  |  |
| 2 years before diagnosis | 1.51 | -0.03, 3.06 |
| 3 months after diagnosis | **2.15** | **0.61, 3.69** |
| 1 year after diagnosis | **3.14** | **1.52, 4.76** |
| 2 years after diagnosis | **2.07** | **0.36, 3.78** |
|  |  |  |
| Overweight |  |  |
| 2 years before diagnosis | **1.52** | **0.03, 3.01** |
| 3 months after diagnosis | 1.37 | -0.11, 2.85 |
| 1 year after diagnosis | **1.97** | **0.40, 3.55** |
| 2 years after diagnosis | 1.06 | -0.57, 2.68 |
|  |  |  |
| **Tumor stage** |  |  |
| Stage I | Reference |  |
| Stage II |  |  |
| 2 years before diagnosis | **2.87** | **1.12, 4.61** |
| 3 months after diagnosis | -0.01 | -1.75, 1.72 |
| 1 year after diagnosis | 1.17 | -0.65, 2.99 |
| 2 years after diagnosis | **2.37** | **0.50, 4.23** |
|  |  |  |
| Stage III |  |  |
| 2 years before diagnosis | **3.77** | **2.23, 5.31** |
| 3 months after diagnosis | -0.13 | -1.65, 1.39 |
| 1 year after diagnosis | 0.97 | -0.68, 2.61 |
| 2 years after diagnosis | **2.85** | **1.10, 4.60** |
|  |  |  |
| **Tumor grade** |  |  |
| Grade 1 | Reference |  |
| Grade 2 |  |  |
| 2 years before diagnosis | 1.69 | -0.16, 3.54 |
| 3 months after diagnosis | 0.18 | -1.67, 2.03 |
| 1 year after diagnosis | -0.29 | -2.21, 1.64 |
| 2 years after diagnosis | 0.45 | -1.55, 2.45 |
|  |  |  |
| Grade 3 |  |  |
| 2 years before diagnosis | **2.58** | **0.40, 4.75** |
| 3 months after diagnosis | 0.97 | -1.20, 3.14 |
| 1 year after diagnosis | 0.93 | -1.37, 3.23 |
| 2 years after diagnosis | 2.11 | -0.28, 4.50 |
|  |  |  |
| Grade 4 |  |  |
| 2 years before diagnosis | **4.59** | **1.64, 7.53** |
| 3 months after diagnosis | 2.62 | -0.33, 5.57 |
| 1 year after diagnosis | **4.74** | **1.49, 8.00** |
| 2 years after diagnosis | **5.85** | **2.50, 9.20** |
|  |  |  |
